# Regulation of Early Plant Development by Red and Blue Light: A Comparative Analysis Between *Arabidopsis thaliana* and *Solanum lycopersicum*

**DOI:** 10.3389/fpls.2020.599982

**Published:** 2020-12-23

**Authors:** Kiki Spaninks, Jelmer van Lieshout, Wim van Ieperen, Remko Offringa

**Affiliations:** ^1^Plant Developmental Genetics, Institute for Biology Leiden, Leiden University, Leiden, Netherlands; ^2^Horticulture and Product Physiology Group, Wageningen University and Research, Wageningen, Netherlands

**Keywords:** tomato, *Arabidopis thaliana*, LED lighting, growth, development, R/B light ratio, floral transition

## Abstract

In vertical farming, plants are grown in multi-layered growth chambers supplied with energy-efficient LEDs that produce less heat and can thus be placed in close proximity to the plants. The spectral quality control allowed by LED lighting potentially enables steering plant development toward desired phenotypes. However, this requires detailed knowledge on how light quality affects different developmental processes per plant species or even cultivar, and how well information from model plants translates to horticultural crops. Here we have grown the model dicot *Arabidopsis thaliana* (Arabidopsis) and the crop plant *Solanum lycopersicum* (tomato) under white or monochromatic red or blue LED conditions. In addition, seedlings were grown *in vitro* in either light-grown roots (LGR) or dark-grown roots (DGR) LED conditions. Our results present an overview of phenotypic traits that are sensitive to red or blue light, which may be used as a basis for application by tomato nurseries. Our comparative analysis showed that young tomato plants were remarkably indifferent to the LED conditions, with red and blue light effects on primary growth, but not on organ formation or flowering. In contrast, Arabidopsis appeared to be highly sensitive to light quality, as dramatic differences in shoot and root elongation, organ formation, and developmental phase transitions were observed between red, blue, and white LED conditions. Our results highlight once more that growth responses to environmental conditions can differ significantly between model and crop species. Understanding the molecular basis for this difference will be important for designing lighting systems tailored for specific crops.

## Introduction

To ensure optimal plant performance in horticultural crops, it is required to understand how growth and development are affected by environmental factors. Light is a key environmental factor that not only affects the available sugars through photosynthesis, but also steers development through processes such as photomorphogenesis, phototropism, and shade avoidance (Nemhauser and Chory, 2002; Goyal et al., 2013; Ballaré and Pierik, 2017). Studies have shown that light intensity can be used to modulate plant growth and ultimately yield in different species (Smeets and Garretsen, 1986; Zhou et al., 2009; Lu et al., 2017; Viršilė et al., 2019). Aside from its intensity, the spectral quality of light influences plant development by activating different families of photoreceptors that can detect light, ranging from UV-B to far-red. Blue light-activated receptor families include cryptochromes (CRYs) (Yu et al., 2010), phototropins (Christie, 2007), and Zeitlupes (ZTLs) (Suetsugu and Wada, 2013), whereas phytochromes (PHYs) respond to red and far-red light (Galvão and Fankhauser, 2015). Many artificial lights that are used in horticulture try to loosely mimic the spectrum of sunlight by including fractions of all the spectral colors. However, the development of LED technology has created new possibilities for spectral control that may lead to more energy efficient and economic lighting. For example, matching the LED spectral output to specific photoreceptor families can ensure optimal plant performance without wasting energy on non-productive wavelengths. Aside from spectral control, LEDs are more energy-efficient than traditional artificial lighting systems and are less detrimental to the environment when discarded, since they contain no toxic metals such as mercury (Morrow, 2008). Finally, LEDs produce less heat and are thus suitable for application in multi-layered vertical farming (SharathKumar et al., 2020).

To implement LED lighting in horticulture it is important to understand how the different colors in the spectrum influence all aspects of plant growth and development. Furthermore, developmental effects of specific LED spectra have been shown to vary between species (Dougher and Bugbee, 2001), suggesting that there are optimal light recipes for different species and even for different ecotypes or cultivars within these species. So far, most studies on spectral properties of light have focused on changes in the red/far-red (R/FR) ratio within the spectrum. At the top of the canopy, R/FR ratios are high, whereas low R/FR fractions are found lower in the canopy (Ballaré et al., 1990). In *Solanum lycopersicum* (tomato), LEDs have been used to add extra far-red light to the spectrum to study shade avoidance (Schrager-Lavelle et al., 2016), plant growth and yield (Ji et al., 2019; Kalaitzoglou et al., 2019) and vitamin production (Ntagkas et al., 2019) among others. Aside from studying R/FR ratios, LED lights can be used to study plant development in response to monochromatic light (red, far-red, yellow, green, or blue) or differential red/blue (R/B) light ratios. So far, most of these studies have been performed in crop species. For example, in tomato, light quality has been found to influence leaf development, assimilates, gas exchange, and biomass (Fan et al., 2013; Lanoue et al., 2017, 2018). However, most of these studies have focused on one crop species, one wavelength, or only on one developmental trait. Moreover, photoreceptor function and downstream pathways have been studied extensively in *Arabidopsis thaliana* (Arabidopsis) (Wang et al., 2016; Lim et al., 2018; Schumacher et al., 2018), but only a small fraction of these pathways have been investigated in commercial crops. In contrast, many light-induced physiological traits have been studied in different crops (Kaiser et al., 2019; Pennisi et al., 2019; Song et al., 2019) but not in Arabidopsis.

Here we performed a comparative analysis between the commercial crop tomato and the genetic model dicot Arabidopsis, studying how monochromatic red or blue LED lighting, compared to white LED lighting, affects early plant development in these species by monitoring several morphological and developmental traits. Although monochromatic red or blue conditions are unlikely to be used in horticulture, this set-up allowed us to obtain more insights into the wavelength-specific effects on plant traits compared to when using different R/B light ratios. Our analyses showed that monochromatic red or blue LED treatments resulted in significant differences in primary growth of both Arabidopsis and tomato, when compared to white LED conditions. However, whereas red and blue light could be used to steer developmental phase transitions in Arabidopsis, in tomato these traits appeared to be surprisingly indifferent to the type of LED treatment. Our results offer an overview of phenotypic traits in young plants that are regulated by red or blue light, and also provide new insights in the conservation and divergence of these traits with respect to their light sensitivity between two plant species from different families.

## Materials and Methods

### Growth Conditions and LED Treatments

In all experiments, plants were grown at a 16 h photoperiod, under white, deep red, or blue Philips Greenpower LED research modules (Signify B.V., Eindhoven, Netherlands) with a measured photon flux density of 120 ± 10 μmol m^–2^s^–1^ at the top of the canopy, a temperature of 21°C, and 70% relative humidity. The percentages of blue, green, red, and far-red wavelengths for the different LED modules are listed in Supplementary Table S1. Experiments with the different LED treatments were performed simultaneously in the same growth chamber in separate compartments enclosed by white plastic screens with a proximal distance of 50 cm to the plants. For *in vitro* analysis of seedling development, two different light treatments were used in all three LED conditions: (1) seedlings were grown completely exposed to light (light-grown roots or LGR); or (2) seedlings were grown in a more “natural” light environment with shoots exposed to light and roots shielded from light using black paper covers (dark-grown roots or DGR) (based on Silva-Navas et al., 2015).

### Plant Lines and Seed Germination

Arabidopsis wild-type ecotypes Columbia (Col-0) and Landsberg *erecta* (L*er*) and tomato cultivar Moneymaker (MM) and the commercial hybrid Foundation (FO) were used in all experiments. This study includes both *in vitro* experiments where seedlings were grown on sterile growth medium as well as experiments where the plants were grown on soil. For *in vitro* experiments, Arabidopsis and tomato seeds were surface sterilized by incubating for 1 min in 70% ethanol and 10 min in a 2-fold diluted commercial bleach solution (1% chlorine). Subsequently the seeds were washed five times with sterile water. Arabidopsis seeds were stratified for 5 days at 4°C in darkness and germinated on square plates (#688102, Greiner Bio-One^TM^) containing MA medium (Masson and Paszkowski, 1992) supplemented with 1% (w/v) sucrose and 0.8% (w/v) Daishin agar. For efficient and simultaneous germination, plates with Arabidopsis seeds were placed vertically in white light for 1 day and then moved to the LED conditions (Supplementary Figure S1A). Sterile tomato seeds were placed on sterilized, wet Whatman filter paper using forceps. Tomato seeds showed optimal germination in darkness (Supplementary Figure S1B) and were therefore kept in darkness at 21°C until 5 days after sowing. Geminated seeds were moved from the filter to square plates containing solid MA medium and placed vertically in the LED conditions. For *on soil* experiments, Arabidopsis seeds were sown on the soil surface and stratified for 5 days at 4°C in darkness. Subsequently the seeds were moved to white light to allow simultaneous germination. After 1 day in white light, the pots were placed in the LED conditions. Tomato seeds were placed approximately 2 cm under the soil surface and pots were directly placed in the LED conditions. The age of tomato plants was therefore expressed as days after sowing (DAS), instead of days after germination (DAG) used for Arabidopsis.

### *In vitro* Analysis of Seedling Development

At 7 days after germination (DAG), Arabidopsis seedlings were photographed, and primary root length and hypocotyl length were measured. Tomato seedlings were photographed at 5 DAG for primary root length and hypocotyl length measurements. All measurements were performed with ImageJ (Fiji) (Schindelin et al., 2012). The shoot-root ratio was calculated based on the measured primary root length and hypocotyl length. At 14 DAG, Arabidopsis seedlings were photographed, and the number of emerged lateral roots was counted using binoculars. Lateral roots could not be counted for tomato since tomato seedlings older than 6 DAG outgrew the square plates.

### Analysis of Leaf Appearance and Morphology

The leaf appearance rate was measured throughout the experiment once or twice per week for tomato and Arabidopsis, respectively. Leaves were counted from the moment they were visible by eye. For Arabidopsis, the plants were grown until bolting. At this time, the rosettes were photographed and rosette surface area (RSA) was measured. Individual rosette leaves were removed and photographed separately for length and width measurements of the leaf blade. Length/width ratio of rosette leaves was calculated based on these measurements. For tomato plants, compound leaves were removed at 45 DAS and photographed individually. Leaf surface area was measured for leaf #4 (fully developed, mature leaf) and leaf #6 (developing, young leaf). All of these measurements were performed with ImageJ (Fiji) (Schindelin et al., 2012).

### Analysis of Flowering Time

Arabidopsis flowering time was measured in number of days until bolting, or until the moment that the first flower buds were visible by eye. For tomato measurements, toothpicks were used to carefully push aside the young leaves from the apex. Flowering time was determined as the day on which small inflorescences became visible near the shoot apex. Individual plants were photographed at 1 week after bolting for Arabidopsis and 30 DAS for tomato.

### Analysis of Stem Development

After Arabidopsis plants became reproductive, plant height measurements commenced. Plant height was measured twice a week until termination of the primary inflorescence meristem. At this time point, individual plants were photographed and the number of branches from the primary inflorescence were counted. Branches were categorized into primary shoots, secondary shoots and tertiary shoots, as previously described (Li et al., 2017). For tomato plants, hypocotyl length, epicotyl length and stem length were measured once a week until 45 DAS. At this time point, individual plants were photographed.

### Statistical Analysis and Figures

All experiments were performed with 20 or 30 biologically independent plants for tomato or Arabidopsis, respectively. For destructive measurements, 10 representative biological replicates were used. Data was obtained from either two or three independent experiments for *on soil* or *in vitro* experiments, respectively. Measurements under different LED conditions, or comparing different ecotypes or cultivars, were statistically analyzed using a one-way ANOVA followed by a Tukey’s honestly significant different (HSD) *post hoc* test. When comparing results from monochromatic (red or blue) with white (control) LED conditions, a two-sided Student’s *t*-test was used. For *in vitro* experiments, LGR and DGR treatments using the same LED condition were also compared using a two-sided Student’s *t*-test. All measurements were plotted into graphs using GraphPad Prism 5 software. In the graphs, the colors of the dots, bars and lines indicate white, red, and blue LED conditions. All photographs were taken with a Nikon D5300 camera and edited in ImageJ (Fiji). Final figures were assembled using Microsoft PowerPoint.

## Results

### Red and Blue Light Influence *in vitro* Development of Arabidopsis and Tomato Seedlings

**FIGURE 1:**
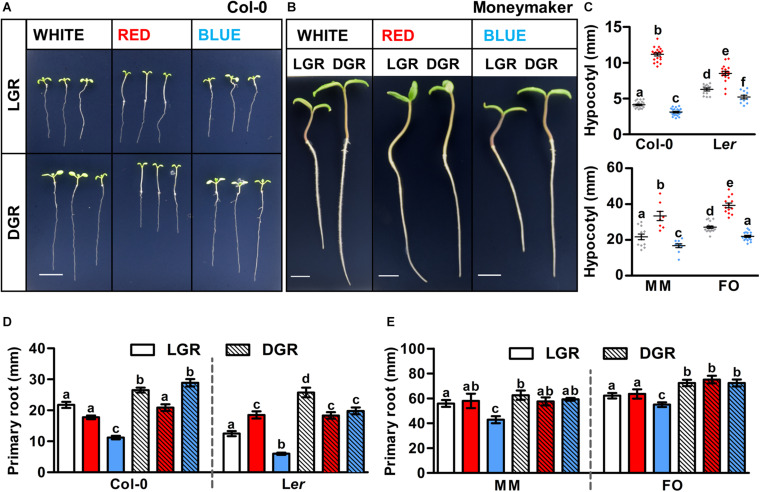
The effect of red and blue light on primary growth of Arabidopsis and tomato seedlings. **(A,B)** Representative 7 day old Arabidopsis and 5 day old tomato seedlings grown in white, red, or blue LED conditions. Seedlings of Arabidopsis ecotype Columbia (Col-0) **(A)** and tomato cultivar Moneymaker (MM) **(B)** were grown in light-grown roots (LGR) and dark-grown roots (DGR) LED conditions. For presentation purposes, seedlings were transferred to black agarose plates before photographing. Scale bars indicate 1 cm. **(C–E)** Quantification of the hypocotyl length of DGR seedlings **(C)** and the primary root length of LGR and DGR seedlings **(D,E)** of Arabidopsis ecotypes Col-0 and Landsberg *erecta* (L*er*) and tomato cultivars MM and Foundation (FO) as shown in (**A,B**, and Supplementary Figures S4A, S5A), respectively. LED conditions and ecotypes or cultivars were compared using a one-way ANOVA followed by a Tukey’s test (letters a–f indicate statistically significant differences values, *p* < 0.05) in **(C–E)**. Error bars represent standard error of the mean in **(C–E)** (*n* = 30). Similar results were obtained in three independent experiments.

Arabidopsis and tomato seedlings were grown in white, red, or blue LED conditions with either light-grown roots (LGR) or dark-grown roots (DGR). Treatment with monochromatic red or blue light strongly affected seedling growth of Arabidopsis ecotypes Col-0 (Figure 1A) and L*er* (Supplementary Figure S4A) and tomato cultivars MM (Figure 1B) and FO (Supplementary Figure S5A). Hypocotyl growth was strongly enhanced in red light and reduced in blue light compared to white light, in both Arabidopsis and tomato seedlings grown either in DGR (Figure 1C) or LGR (Supplementary Figures S2A,C) conditions, making it the most conserved trait regulated by light quality. Red- or blue light-induced alterations of primary root growth were only partially conserved between the two species. In both Arabidopsis (Figure 1D) and tomato (Figure 1E), seedlings grown in monochromatic blue LGR conditions had shorter roots than in white LGR conditions, whereas there was no difference between blue and white DGR conditions (with the exception of L*er* DGR seedlings). This suggests that blue light inhibits root growth locally, and not through shoot-to-root signaling. In monochromatic red light, Arabidopsis, but not tomato seedlings, showed reduced primary root growth compared to white light in DGR conditions, but not in LGR conditions (Figures 1D,E), suggesting that in Arabidopsis red LED conditions hamper root growth by shoot-to-root signaling. In conclusion, our results show that *in vitro* growth of both Arabidopsis and tomato seedlings can be altered by light quality. The local effect of light quality on primary root, and hypocotyl growth seems conserved between these two species, whereas the effect of light quality mediated by shoot-to-root signaling seems more species- or cultivar-dependent. In addition, our results suggest that light conditions with higher rather than lower R/B ratios, and dark-grown roots are optimal for *in vitro* seedling development.

### Red Light Promotes Shoot Elongation in Arabidopsis and Young Tomato Plants

**FIGURE 2:**
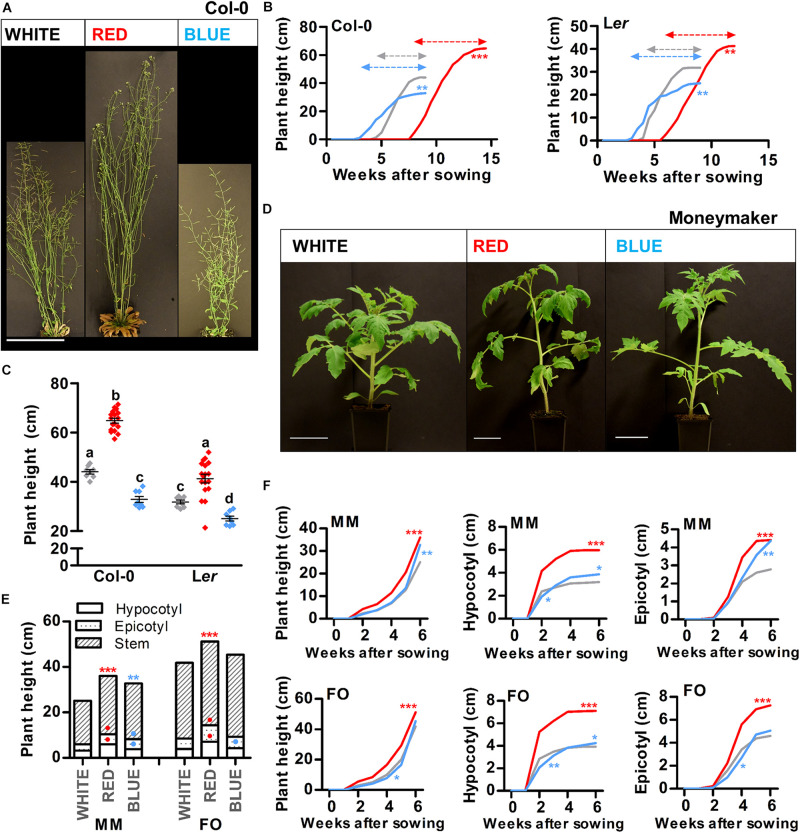
Red light promotes shoot growth in Arabidopsis and young tomato plants. **(A)** Representative Arabidopsis Columbia (Col-0) plants grown in white, red, or blue LED conditions until 4 weeks after bolting. **(B,C)** Quantification of the plant height over time **(B)** or the plant height after termination of the primary inflorescence **(C)** of Arabidopsis Col-0 or Landsberg *erecta* (L*er*) plants as shown in (**A** and Supplementary Figure S4E), respectively. **(D)** Representative tomato Moneymaker (MM) plants grown in white, red, or blue LED conditions until 45 days after sowing (DAS). **(E,F)** Quantification of the plant height at 45 DAS **(E)**, or the plant height, hypocotyl length, or epicotyl length over time **(F)** of tomato MM or Foundation (FO) plants as shown in (**D** and Supplementary Figure S5D), respectively. LED conditions and ecotypes or cultivars were compared using a one-way ANOVA followed by a Tukey’s test (letters a–d indicate statistically significant differences, *p* < 0.05) in **(C)**. In **(B,E,F)**, monochromatic LED conditions (red or blue) were compared to white (control) using a two-sided Student’s *t*-test (asterisks indicate significant differences (*p* < 0.05) in time series in **(B,F)**, or in plant height in **(E)**, bullets indicate significant differences (*p* < 0.05) in hypocotyl or epicotyl length in **(E)**. Error bars represent standard error of the mean in **(C),** standard errors for **(B,E,F)** are listed in Supplementary Table S2 (*n* = 20). Dashed arrows in **(B)** represent the time from bolting until termination of the primary inflorescence. For presentation purposes, pots were placed in front of a black background in **(A,D)** before photographing. Scale bars indicate 10 cm in **(A)**, and 5 cm in **(D)**. Similar results were obtained in two independent experiments.

The height of a plant determines its ability to compete for light and therefore often correlates with leaf mass, seed production and longevity among others (Moles et al., 2009). For monopodial species such as Arabidopsis, stem growth is initiated once the plant becomes reproductive and continues until termination of the inflorescence meristems (IMs) (Schmitz and Theres, 1999). To investigate if shoot elongation can be modulated by light quality, Arabidopsis plants were grown in white, red, or blue LED conditions, until termination of the primary IM (Col-0: Figure 2A and L*er*: Supplementary Figure S4E). At this time, plant height of Col-0 and L*er* ecotypes was significantly reduced in blue light and increased in red light, compared to white light (Figure 2C). In a series of weekly measurements, we observed that the primary IM of plants grown in monochromatic blue or red light produced flowers for approximately 6 weeks, whereas in white light grown plants the primary IM terminated after approximately 5 weeks (Figure 2B, dashed arrows). This slight extension of the reproductive phase in blue light compared to white light, indicated that the reduction of plant height in blue light is caused by reduced elongation of the shoot, and not by a shorter growth phase. In contrast, the elongated plants in red light might be caused by both enhanced elongation growth, and the extended reproductive phase, when compared to white light. As a sympodial plant, tomato initiates stem growth already during the vegetative growth phase (Schmitz and Theres, 1999). To investigate shoot elongation of tomato plants grown in white, red, or blue LED conditions, we measured hypocotyl length, epicotyl length and stem length (from epicotyl to SAM) every week for up to 45 days after sowing (DAS). At 45 DAS, red light grown plants of both cultivars were taller than white light grown plants (MM and FO: Figures 2D–F and Supplementary Figure S5D). Also at earlier timepoints, tomato plants grown in red light had a significantly longer hypocotyl, epicotyl, and stem than white light grown plants (Figure 2F). At 45 DAS, MM plants grown in blue light were significantly taller than those grown in white light (Figures 2D,E), whereas FO plants only showed a significant increase in hypocotyl length in blue light (Figure 2E and Supplementary Figure S5D). However, during our weekly measurements we observed that, at earlier time points (mainly before the appearance of inflorescence meristems), blue light grown plants of both cultivars had shorter hypocotyls, epicotyls and stems compared to white light grown plants (Figure 2F). This shows that, in tomato, the effects of monochromatic blue light treatment on shoot elongation are dependent on both cultivar and developmental stage. Taken together, our results show that the enhanced shoot elongation in monochromatic red LED conditions is conserved between Arabidopsis and tomato, whereas the effect of monochromatic blue light seems to vary between species and cultivars.

### Monochromatic Red Light Promotes Shoot Growth and Inhibits Root Branching in Arabidopsis

**FIGURE 3:**
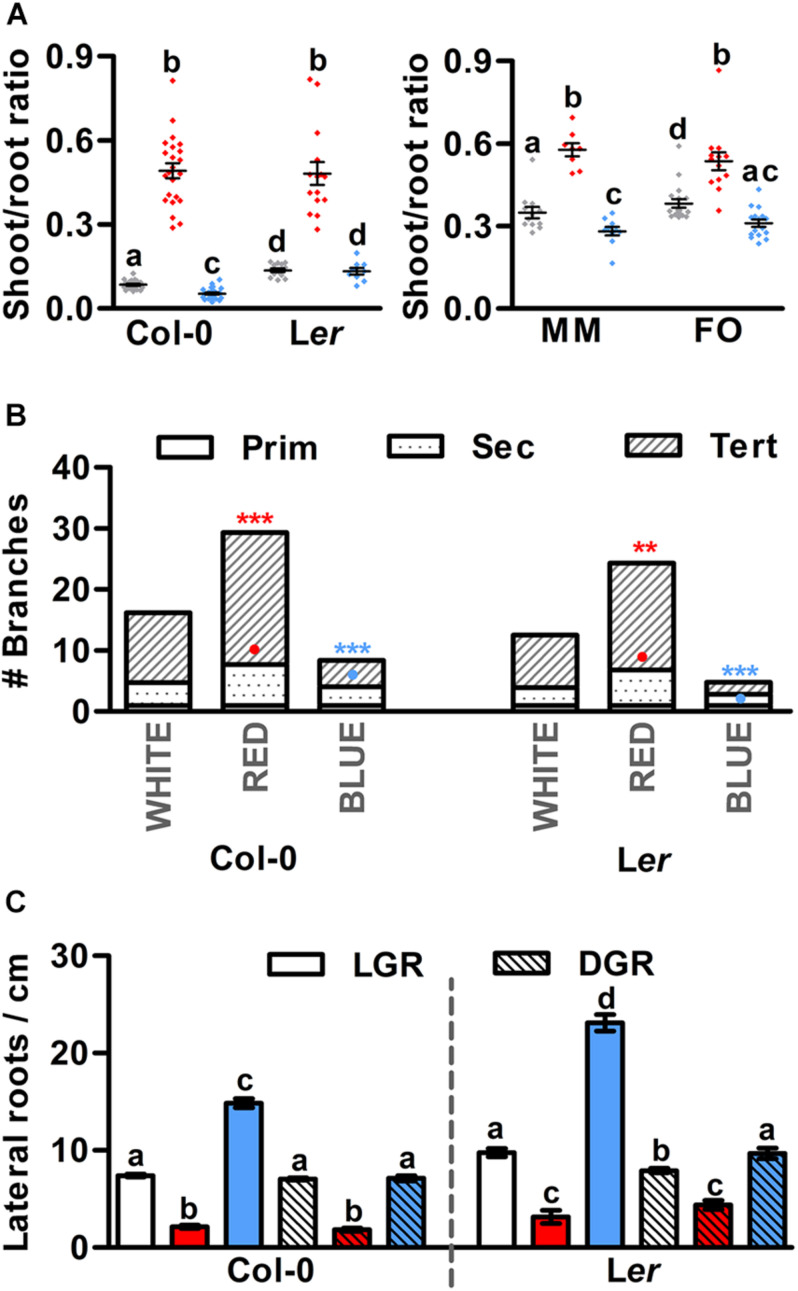
Monochromatic red light promotes shoot growth and inhibits root branching in Arabidopsis. **(A)** Shoot-root ratio of 7 day old Arabidopsis seedlings (left) and 5 day old tomato seedlings (right), grown in white, red, or blue LED conditions. Arabidopsis ecotypes Columbia (Col-0) and Landsberg *erecta* (L*er*), and tomato cultivars Moneymaker (MM) and Foundation (FO) were grown in dark-grown roots (DGR) LED conditions. **(B)** Number of primary (Prim), secondary (Sec) and tertiary (Tert) branches from the primary inflorescence of Arabidopsis Col-0 and L*er* plants grown in LED conditions until termination of the primary inflorescence. **(C)** Lateral root density of 14-day old Col-0 and L*er* seedlings grown in light-grown roots (LGR) and DGR LED conditions. Graph colors represent the LED conditions in **(A,C)**. LED conditions and ecotypes or cultivars were compared using a one-way ANOVA followed by a Tukey’s test (letters a–d indicate statistically significant differences, *p* < 0.05) in **(A**,**C)**. In **(B)**, monochromatic LED conditions (red or blue) were compared to white (control) using a two-sided Student’s *t*-test [bullets indicate significant differences in secondary branches (*p* < 0.05), asterisks indicate significant differences in tertiary branches (***p < 0.001, **p < 0.01)]. Error bars represent standard error from mean in **(A,C)** (*n* = 30), standard errors for **(B)** are listed in Supplementary Table S2 (*n* = 20). Similar results were obtained in three **(A,C)** or two **(B)** independent experiments.

In nature, the balance between shoot growth to increase photosynthetic capacity, and root growth to compete for soil nutrients is tightly controlled and dependent on the growth conditions and nutrient and water availability (Puig et al., 2012). In greenhouses, however, the growth conditions and availability of water and nutrients are generally good, making development of the root system less relevant. As a result, plant breeders of fruit-producing species have spent decades to optimize the growth and development of above-ground organs (Van der Ploeg et al., 2007), often at the cost of root development. In our *in vitro* experiments, monochromatic red conditions, either LGR or DGR, significantly enhanced the shoot-root ratio of both Arabidopsis and tomato seedlings (Figure 3A and Supplementary Figures S2B,D). A mildly opposite effect was observed in seedlings grown under monochromatic blue LED DGR conditions (Figure 3A). In LGR conditions, however, Arabidopsis seedlings showed a slightly increased shoot-root ratio (Supplementary Figures S2B,D), which is most likely the result of the strong local inhibition of primary root growth in monochromatic blue light (Figures 1D,E). This suggests that the balance between shoot and root elongation in Arabidopsis and tomato seedlings can be controlled by the R/B light ratio in the spectrum. Interestingly, analysis of the number of branches on the primary Arabidopsis inflorescence showed that bud formation from axillary meristems is greatly enhanced in red light compared to white light conditions (Figure 3B). In contrast, red light grown Arabidopsis seedlings showed a significant decrease in lateral root density compared to those grown in white light, in both LGR and DGR conditions (Figure 3C and Supplementary Figure S3). In monochromatic blue light, branching of the primary inflorescence was significantly reduced compared to white light (Figure 3B). The lateral root density of blue light DGR Arabidopsis seedlings was unaffected (Figure 3C and Supplementary Figure S3), but was increased in LGR seedlings, most likely as a result of primary root growth inhibition in blue LGR conditions (Figures 1D,E, 3C). To summarize, our results show that Arabidopsis plants grown in monochromatic red LED conditions show increased shoot elongation and branching, and decreased root branching compared to white light grown plants. In contrast, the effect of monochromatic blue light is relatively mild, except for the strong inhibitory effect on root growth in LGR conditions. Tomato plants show the same increased shoot-root ratio in monochromatic red compared to white light, and a similar mild effect of monochromatic blue light, but the effects of red light on lateral organ formation in tomato shoots and roots remain to be determined.

### Developmental Phase Transitions in Arabidopsis Are Promoted by Blue Light and Delayed by Red Light

**FIGURE 4:**
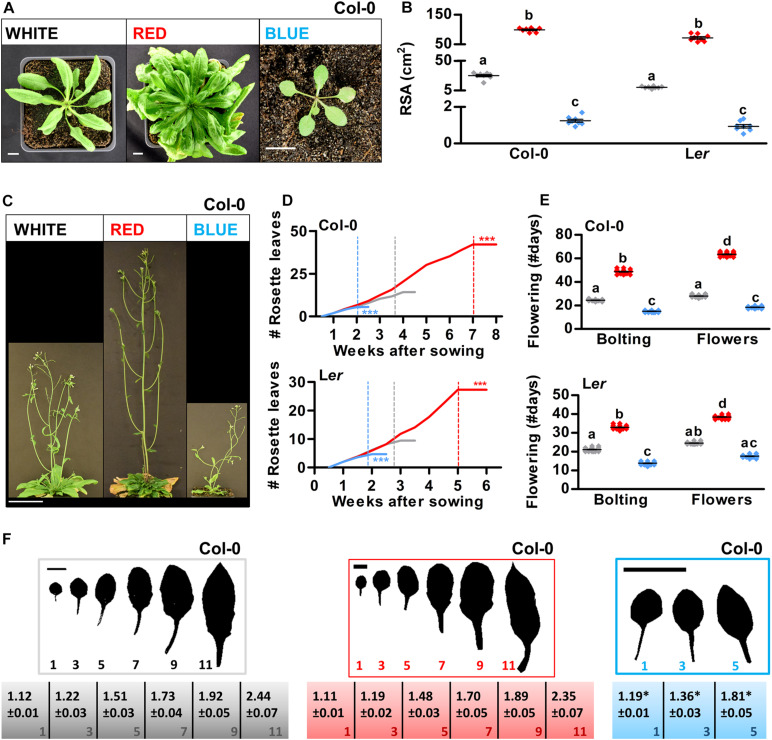
Developmental phase transitions in Arabidopsis are promoted by blue light and delayed by red light. **(A)** Rosette phenotype of representative Arabidopsis plants of ecotype Columbia (Col-0) grown in white, red, or blue LED conditions. **(B)** Quantification of rosette surface area (RSA) of Col-0 or Landsberg *erecta* (L*er*) plants as shown in (**A** and Supplementary Figure S4B), respectively. **(C)** Representative Arabidopsis Col-0 plants grown in LED conditions until 1 week after flowering. **(D)** Rosette leaf appearance in Col-0 and L*er* plants over time. **(E)** Flowering time (until bolting, or until the appearance of flower buds) of Col-0 and L*er* plants in number of days. **(F)** Rosette leaves of representative Col-0 plants and length/width ratios of the leaf blade (± SE, *n* = 10). Scale bars represent 1 cm in **(A**,**F)**, and 10 cm in **(C)**. Graph colors represent the LED conditions in **(B,D,E)**. LED conditions and ecotypes were compared using a one-way ANOVA followed by a Tukey’s test (letters a–d indicate statistically significant differences, *p* < 0.05) in **(B,E)**. In **(D,F)**, monochromatic LED conditions (red or blue) were compared to white (control) using a two-sided Student’s *t*-test [asterisks indicate significant differences (*p < 0.05, ***p < 0.001)]. Error bars represent standard error of the mean in **(B,E)** (*n* = 30), standard errors for **(D)** are listed in Supplementary Table S2 (*n* = 30). Dashed lines in **(D)** represent the time of bolting. Similar results were obtained in two independent experiments.

To ensure a high yield in crops, it is important that leaves are produced at an optimal rate and that the morphology of the leaf allows for optimal exposure to light (Mathan et al., 2016). Moreover, optimizing the timing of flowering is crucial to ensure either a long vegetative phase (for leaf production in crop species such as lettuce or cabbage) or a short vegetative phase (for rapid breeding cycles or for fruit-producing species such as tomato). Previous studies that used light filters or continuous lighting indicated that developmental phase transitions in Arabidopsis can be modulated by light quality (Eskins, 1992; Guo et al., 1998). To investigate if similar phenotypes could be obtained using a LED setup with a 16/8 h day/night cycle, Arabidopsis plants were grown on soil in white, red, or blue LED conditions. In monochromatic blue light, the rosette size, expressed as rosette surface area (RSA), was greatly reduced, whereas white light grown plants showed a regular rosette development, and monochromatic red light grown plants developed large rosettes resembling those of Arabidopsis plants grown in short-day conditions (Figures 4A,B and Supplementary Figure S4B; Brandt et al., 2018). Both the increase of RSA in red LED conditions and the decrease of RSA in blue LED conditions correlated with significant changes in the timing of the plant’s floral transition (Col-0: Figures 4C–E and L*er*: Supplementary Figure S4D). Col-0 and L*er* plants that were grown in blue light produced a limited number of rosette leaves as they flowered extremely early, whereas plants that were grown in red light developed many rosette leaves during an extended vegetative phase due to late flowering (Figures 4D,E). In Arabidopsis, the floral transition is preceded by the juvenile-to-adult or vegetative phase transition, the occurrence of which can be determined by leaf heteroblasty. Juvenile leaves consist of a round leaf blade with a long petiole, with a length/width ratio of approximately 1, whereas adult leaves have a more serrated leaf blade with a short petiole, and with a length/width ratio of approximately 1.7 (Telfer et al., 1997). Based on their length/width ratio, leaves of blue light grown plants seemed to mature significantly faster, although in L*er*, no completely adult leaves were formed before the plants switched to the reproductive phase (Col-0: Figure 4F and L*er*: Supplementary Figure S4C). In red light grown plants, the timing of the vegetative phase changes did not differ significantly from that of white light grown plants, suggesting that, in contrast to the reproductive phase transition, the vegetative phase transition was not delayed by the monochromatic red light treatment. Altogether, our results show that especially the floral transition but also the vegetative phase transition in Arabidopsis are sensitive to light quality and can thus be modulated not only by day length but also by the R/B light ratio in the spectrum.

### Developmental Phase Transitions in Tomato Are Indifferent to the R/B Light Ratio

**FIGURE 5:**
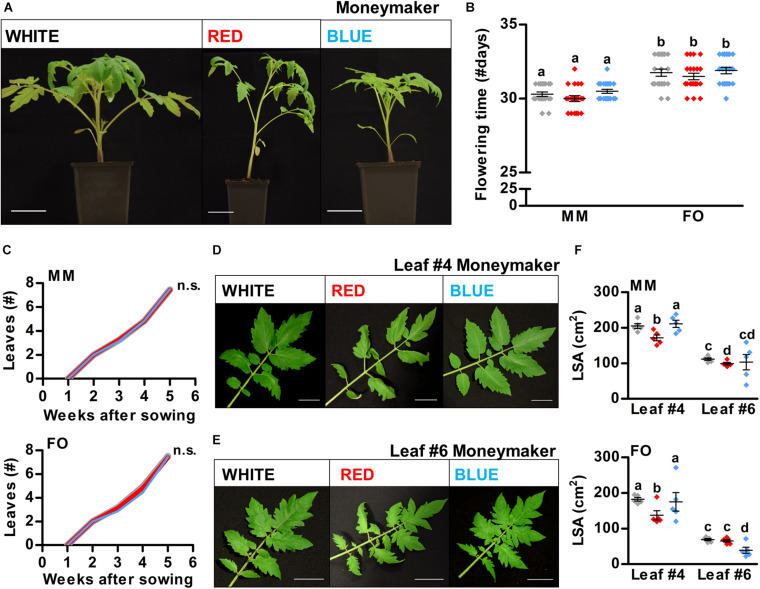
Developmental phase transitions in tomato are indifferent to R/B light ratios. **(A)** Representative tomato plants of cultivar Moneymaker (MM) grown in white, red, or blue LED conditions until 30 days after sowing (DAS). **(B)** Flowering time of MM and Foundation (FO) plants in number of days. **(C)** Leaf appearance over time in MM and FO plants. **(D,E)** Representative compound leaves from MM plants grown in LED conditions until 45 DAS: leaf #4 **(D)** and leaf #6 **(E)**. For presentation purposes, leaves were removed, flattened, and placed on black paper. **(F)** Quantification of leaf surface area (LSA) of MM and FO leaves shown in (**D,E**, and Supplementary Figures S5B,C), respectively. Scale bars represent 5 cm in **(A,D,E)**. Graph colors represent the LED conditions in **(B,C,F)**. LED conditions and cultivars were compared using a one-way ANOVA followed by a Tukey’s test (letters a–d indicate statistically significant differences, *p* < 0.05) in **(B,F)**. In **(C)**, monochromatic LED conditions (red or blue) were compared to white (control) using a two-sided Student’s *t*-test [n.s. indicates no significant differences between LED conditions (*p* < 0.05)]. Error bars represent standard error of the mean in **(B**,**F)** (*n* = 20), standard errors for **(C)** are listed in Supplementary Table S2 (*n* = 20). Similar results were obtained in two independent experiments.

To investigate if developmental phase transitions can be modulated by red and blue light in tomato as well, MM and FO plants were grown on soil in white, red, or blue LED conditions until the start of the reproductive phase, which was defined as the moment that the first inflorescences appeared near the shoot apex (Figure 5A). MM and FO plants became reproductive at approximately 30 and 32 DAS, respectively, in all three LED conditions (Figure 5B). In addition, the appearance rate of new compound leaves was the same in all three LED conditions and in both cultivars (Figure 5C). These results are in contrast to our observations in Arabidopsis and imply that developmental phase shifts in tomato are completely indifferent to the R/B light ratio. To investigate the sensitivity of tomato leaf morphology to red and blue light, MM and FO plants were grown in the three different LED conditions until 45 DAS. We used leaf #4 as a representative for fully developed leaves (MM: Figure 5D and FO: Supplementary Figure S5B), and leaf #6 as a representative for young, not fully developed leaves (MM: Figure 5E and FO: Supplementary Figure S5C) for leaf surface area (LSA) measurements. The LSA of leaf #4 was similar for plants grown in white and blue LED conditions (Figure 5F). However, leaf #6 of blue light grown FO plants showed a decreased LSA, which is most likely a result of a slight delay in leaf development specific for this cultivar, and not a true effect of monochromatic blue light on leaf morphology. In contrast, monochromatic red LED conditions led to a significant decrease in LSA of leaf #4 in both cultivars (Figure 5F). Moreover, leaves of plants grown in red light showed epinasty (Figures 5D,E and Supplementary Figures S5B,C), thus further reducing the effective LSA for photosynthesis. In conclusion, light quality does have an effect on leaf morphology, and may alter photosynthetic capacity in tomato. However, these changes in leaf morphology do not influence the formation rate of new leaves or flowering time. Although developmental phase transitions in Arabidopsis are highly sensitive to light quality, to our surprise the same phase transitions in tomato appeared to be completely indifferent to the R/B light ratio.

## Discussion

Recent developments in LED technology have created new possibilities for spectral control that allow us to use light quality to steer plant development (Morrow, 2008). Here we present an overview of the phenotypes that arise from growing Arabidopsis and young tomato plants in white or monochromatic red or blue LED lighting. During *in vitro* seedling development, hypocotyls were significantly more elongated in red light and shorter in blue light, compared to white light grown Arabidopsis and tomato seedlings. This confirmed previously published results that were obtained with the use of light filters (Ballaré et al., 1995), or with lighting setups in which the light intensity differed greatly between LED conditions (Jensen et al., 1998). At later developmental stages, Arabidopsis and tomato plant height were significantly increased in monochromatic red light and decreased in monochromatic blue light. In tomato, however, the reduced plant elongation in monochromatic blue light was limited to early stages of plant development. These results are in line with previous studies in wheat (Monostori et al., 2018) and chili peppers (Gangadhar et al., 2012), and a recent greenhouse study in tomato where LEDs were used as supplemental lighting (Dieleman et al., 2019). However, monochromatic blue light has been reported to enhance hypocotyl growth in cucumber, indicating that there are species-specific differences (Hernández and Kubota, 2016). Primary shoot growth in white light grown seedlings and plants was intermediate between that in monochromatic red or blue light grown seedlings and plants, suggesting an antagonistic effect of both light conditions, with red light promoting and blue light inhibiting shoot growth. Since auxin, ethylene, gibberellic acid and brassinosteroids are the main phytohormones that regulate hypocotyl and stem elongation in response to light (Vandenbussche et al., 2005; Kurepin and Pharis, 2014), it is likely that red- and blue light-responsive photoreceptors interact with the corresponding hormone signaling pathways. We also observed a significant effect of red and blue light on primary root growth in Arabidopsis and tomato seedlings. By combining the different LED conditions with LGR (light-grown roots) and DGR (dark-grown roots) conditions, we were able to show that the reduced primary root growth in monochromatic blue light is caused by a local light-induced inhibition of root growth. As auxin and cytokinin are the main regulators of primary root growth (Su et al., 2011), we expect that activation of root-localized photoreceptors affects cytokinin levels and auxin gradients in the root apical meristem. In contrast, we observed reduced primary root growth in Arabidopsis seedlings grown in red DGR, but not LGR conditions, suggesting that red LED conditions inhibit root growth by altering the shoot to root signaling. In this case, we expect that activation of shoot-localized photoreceptors influences shoot to root transport of key signaling molecules such as HY5, HYH or auxin to modulate primary root growth (Chen et al., 2016; Van Gelderen et al., 2018). To summarize, our results show that primary growth of Arabidopsis and tomato can be modulated by changing the light quality at different developmental stages, and in different ecotypes or cultivars (Table 1). In this way, light quality may be used to steer primary growth toward compact and sturdy crop plants which can be grown in multi-layered growth chambers.

**TABLE 1:** Primary growth of Arabidopsis and tomato is regulated by red and blue light.

	**Arabidopsis**		**Tomato**	
	**Red**	**Blue**	**Red**	**Blue**
Primary root growth	*Similar to W	Shorter root	Similar to W	Shorter root
Hypocotyl length	Longer hypocotyl	Shorter hypocotyl	Longer hypocotyl	Shorter hypocotyl
Shoot/root ratio	Higher S/R ratio	*Similar to W	Higher S/R ratio	Similar to W
Epicotyl length	N/A	N/A	Longer epicotyl	*Shorter epicotyl
Plant height	Taller plants	Shorter plants	Taller plants	**Shorter/taller plants

*Summary of the Arabidopsis and tomato primary growth phenotypes that were induced by monochromatic red or blue light in LGR conditions. Statistically significant differences between white light (control) and monochromatic LED conditions (red or blue) are indicated in this table (p < 0.05). When no statistical differences were found between LED conditions, it is indicated as “similar to white (W).” Asterisks indicate results that are ecotype-, or cultivar-dependent. Double Asterisks indicate results that are time-dependent.*

In Arabidopsis, we observed a considerable increase in the shoot-root ratio in monochromatic red light, and a slight decrease in the shoot-root ratio in monochromatic blue light, which resulted from light-induced changes in hypocotyl growth and, to a lesser extent, primary root growth. Moreover, the lateral organ density in roots was greatly decreased in red LED conditions. Since the far-red light-activated phytochrome A has been shown to promote lateral root formation (Salisbury et al., 2007), it is likely that the low number of lateral roots in monochromatic red light results from red light-inactivation of this photoreceptor. Previous studies have shown that blue light photoreceptors suppress lateral root formation (Zeng et al., 2010; Moni et al., 2015). In contrast, we observed an increase in lateral root density in monochromatic blue light. We suspect that the strong decrease in primary root growth in blue LED conditions is responsible for an indirect increase in lateral root density similar to in white LED conditions. In contrast to the roots, shoot branching was significantly enhanced in monochromatic red light, and significantly decreased in monochromatic blue light, whereas white light grown plants showed an intermediate phenotype. Shoot branching is promoted by cytokinin, and inhibited by strigolactones, either directly or through interactions with auxin (Domagalska and Leyser, 2011; Brewer et al., 2013). This suggests that red light might either enhance cytokinin signaling, or inhibit strigolactones, to promote shoot branching, and that an opposite effect on these phytohormones might be expected for blue light. This hypothesis is in line with previous studies that show that the blue light photoreceptor cryptochrome 1 inhibits shoot branching, and that the red light-inducible phytochrome B promotes shoot branching through auxin signaling (Reddy and Finlayson, 2014; Zhai et al., 2020). Although we demonstrate that the balance between shoot and root development can be steered by the light quality in Arabidopsis, additional research is required for horticultural application.

Our comparative analysis identified a remarkable difference in the regulation of developmental phase transitions by light quality between Arabidopsis and tomato. We observed that Arabidopsis plants grown in monochromatic blue light developed very small rosettes and flowered early, whereas plants grown in monochromatic red light developed extremely big rosettes due to late flowering. Our results confirm previous studies in which light filters were used, or where plants were grown under continuous LED illumination, which excludes the effect of day-length (Eskins, 1992; Guo et al., 1998). The light-induced changes in leaf length/width ratios, leaf formation and RSA in Arabidopsis are most likely the result of light quality-induced changes in both the juvenile to adult vegetative and the adult vegetative to reproductive phase transition (also referred to as the vegetative phase change and the floral transition, respectively). Strikingly, in contrast to Arabidopsis, these phase transitions in tomato were completely indifferent to red and blue light (Table 2). This might be a result of fundamental differences in plant architecture (monopodial vs. sympodial growth), daylength sensitivity (long-day vs. day-neutral) or life history (annual vs. semi-perennial) between Arabidopsis and tomato, respectively. Similar to the phenotypes that we observed in Arabidopsis, strawberry and petunia have been shown to flower early in blue light and late in red light (Fukuda et al., 2012; Fukuda et al., 2016; Yoshida et al., 2016). Petunia and tomato are both members of the *Solanaceae* family and are categorized as sympodial, semi-perennial plants. However, in contrast to tomato, petunia is not a day-neutral plant but a long-day plant, suggesting that photoperiodic sensitivity is a key characteristic of plants for which developmental phase transitions are sensitive to red or blue light. Because Arabidopsis plants grown in white light show an intermediate phenotype compared to those grown in either monochromatic red or blue LED conditions, a separate phase transition-promoting effect of blue light and a phase transition-delaying effect of red light should be considered. Previous studies have shown that blue light promotes flowering through photoreceptors of the cryptochrome and Zeitlupe families. In response to blue light, these photoreceptors enhance expression of *CONSTANS* (*CO*). As a main integrator of circadian clock components and light signaling, CO promotes flowering through the florigen *FLOWERING LOCUS T*, in response to day length (Valverde, 2011). In day-neutral plant species, components of the photoperiodic pathway are likely non-existent, or unresponsive (Mizoguchi et al., 2007), which might explain the indifference of tomato plants to LED conditions that lack blue light. Although red light has been shown to inhibit flowering through targeted degradation of CO proteins (Lazaro et al., 2015), we do not expect that the flower-delaying effect of red light relies solely on photoperiodicity. Based on the length/width ratios of leaf blades, we suggest that meristems of plants grown in monochromatic blue light may mature faster, whereas meristems of plants grown in monochromatic red light mature at the same rate as those in white light. This suggests that red light might inhibit the aging pathway, in addition to the photoperiodic pathway, to delay the floral transition. Therefore, LED conditions that lack red light would result in an early vegetative phase transition and early flowering. To summarize, our observations in Arabidopsis suggest a possibility to identify more (long-day) species in which developmental phase transitions can be steered by light quality, whereas our experiments in tomato demonstrate that tomato growers may change the R/B light ratio toward desired phenotypes, without affecting the timing of the developmental phase transitions. If we wish to apply the R/B light ratio to steer the timing of developmental phase transitions in horticulture, it will be necessary to further investigate the LED phenotypes in Arabidopsis, and to verify whether these are conserved in other species from the same or from different families. However, changes in the LED spectrum are likely to simultaneously modulate the activity of multiple photoreceptors, and the interplay between photoreceptors and their downstream targets adds another layer of complexity. For example, it has been shown that blue light-activated cryptochromes physically interact with the far-red/red light-inducible phytochromes, and with their downstream targets (Mas et al., 2000; Pedmale et al., 2016). Nonetheless, identification of the key photoreceptors, phytohormones, and downstream signaling targets that underly the phenotypes that we observed in this study will be the next step toward optimizing light quality-induced phenotypic traits for horticultural application, and to understand the divergence of these traits between plant species.

**TABLE 2:** Developmental phase transitions are modulated by red and blue light in Arabidopsis, but not in tomato.

	**Arabidopsis**		**Tomato**	
	**Red**	**Blue**	**Red**	**Blue**
Leaf formation	More leaves	Less leaves	Similar to W	Similar to W
Leaf morphology	Bigger leaves/bigger rosette	Smaller leaves/smaller rosette	Smaller leaves	Similar to W
Flowering time	Late	Early	Similar to W	Similar to W

*Summary of the Arabidopsis and tomato developmental phenotypes that were induced by monochromatic red or blue light. Statistically significant differences between white light (control) and monochromatic LED conditions (red or blue) are indicated in this table (p < 0.05). When no statistical differences were found between monochromatic light and white light, it is indicated as “similar to white (W).”*

## Conclusion

Our results demonstrate that light quality modulates different aspects of the growth and early development of Arabidopsis and tomato. In Arabidopsis, treatment with monochromatic red light resulted in increased shoot growth and development (sometimes at the cost of root development), and delayed flowering, whereas plants grown in monochromatic blue light showed reduced shoot growth and development, and early flowering. In tomato plants grown in monochromatic red light we observed increased shoot growth and development, and a decrease in leaf surface area, whereas tomato plants grown in blue LED conditions showed reduced shoot growth in vegetative plants and increased shoot growth in flowering plants. Our comparative analysis showed that most of the primary growth responses to light quality were conserved between Arabidopsis and tomato (Table 1). In contrast, developmental phase transitions in Arabidopsis were highly sensitive to light quality, whereas these transitions in tomato were completely indifferent to red and blue light (Table 2).

## Data Availability Statement

The original contributions presented in the study are included in the article/Supplementary Material, further inquiries can be directed to the corresponding author/s.

## Author Contributions

KS, WI, and RO conceived and designed the experiments and analyzed the results. KS and JL performed the experiments. KS performed the statistical analysis. KS and RO wrote the manuscript. All authors contributed to manuscript revision and read and approved the submitted version.

## Conflict of Interest

The authors declare that the research was conducted in the absence of any commercial or financial relationships that could be construed as a potential conflict of interest.
