# Supplementary material for: Regulation of Early Plant Development by Red and Blue Light: A Comparative Analysis Between Arabidopsis thaliana and Solanum lycopersicum
Source: Front Plant Sci. 2020 Dec 23;11:599982. doi: 10.3389/fpls.2020.599982 (PMC7785528; doi:10.3389/fpls.2020.599982)
Supplement: Supplementary file 1 [file Data_Sheet_1.PDF]

Supplementary data (Tables S1-S2 and Figures S1-S5)

Table S1: LED modules

This table indicates the percentages of blue, green, red, and far-red wavelengths for the three types of LED modules that were used in this study.

COLOR	WAVELENGTHS	WHITE LEDS	RED LEDS	BLUE LEDS
BLUE	400-499	58%	0%	100%
GREEN	500-599	25%	0%	0%
RED	600-699	15%	100%	0%
FAR-RED	700-799	2%	0%	0%

Table S2: Standard error values

In some figures, error bars were not included in the graphs for presentation purposes. These standard error values are listed in the table below. Abbreviations that are used: Arabidopsis (Ara), Tomato (Tom), Columbia (Col), Landsberg *erecta* (Ler), Moneymaker (MM), Foundation (FO), weeks after sowing (WAS), and days after sowing (DAS).

FIGURE	DATA POINT	MEAN VALUE	STANDARD ERROR
2B	Col-White-1WAS	0	0
2B	Col-White-2WAS	0	0
2B	Col-White-3WAS	0	0
2B	Col-White-4WAS	1.3	0.10
2B	Col-White-5WAS	12.8	1.78
2B	Col-White-6WAS	29.3	2.15
2B	Col-White-7WAS	40.7	3.62
2B	Col-White-8WAS	44.1	3.89
2B	Col-White-9WAS	44.2	3.91
2B	Col-Blue-1WAS	0	0
2B	Col-Blue-2WAS	0	0
2B	Col-Blue-3WAS	4.7	0.32
2B	Col-Blue-4WAS	13.9	1.63
2B	Col-Blue-5WAS	21.6	2.09
2B	Col-Blue-6WAS	29.2	2.74
2B	Col-Blue-7WAS	31.3	3.68
2B	Col-Blue-8WAS	32.6	3.77
2B	Col-Blue-9WAS	32.9	3.81
2B	Col-Red-1WAS	0	0
2B	Col-Red-2WAS	0	0
2B	Col-Red-3WAS	0	0
2B	Col-Red-4WAS	0	0
2B	Col-Red-5WAS	0	0
2B	Col-Red-6WAS	0	0
2B	Col-Red-7WAS	0	0
2B	Col-Red-8WAS	10.6	0.51
2B	Col-Red-9WAS	26.4	1.77

2B	Col-Red-10WAS	41.5	3.49
2B	Col-Red-11WAS	53.2	4.21
2B	Col-Red-12WAS	60.1	4.87
2B	Col-Red-13WAS	63.8	5.02
2B	Col-Red-14WAS	64.8	5.11
2B	Ler-White-1WAS	0	0
2B	Ler-White-2WAS	0	0
2B	Ler-White-3WAS	0	0
2B	Ler-White-4WAS	9.5	0.61
2B	Ler-White-5WAS	19.3	1.84
2B	Ler-White-6WAS	26.9	2.33
2B	Ler-White-7WAS	31.6	2.98
2B	Ler-White-8WAS	31.8	3.10
2B	Ler-White-9WAS	31.9	3.08
2B	Ler-Blue-1WAS	0	0
2B	Ler-Blue-2WAS	0	0
2B	Ler-Blue-3WAS	5.1	0.32
2B	Ler-Blue-4WAS	15.0	1.13
2B	Ler-Blue-5WAS	19.3	1.87
2B	Ler-Blue-6WAS	21.1	1.93
2B	Ler-Blue-7WAS	23.7	2.16
2B	Ler-Blue-8WAS	24.8	2.39
2B	Ler-Blue-9WAS	25.1	2.38
2B	Ler-Red-1WAS	0	0
2B	Ler-Red-2WAS	0	0
2B	Ler-Red-3WAS	0	0
2B	Ler-Red-4WAS	0	0
2B	Ler-Red-5WAS	0	0
2B	Ler-Red-6WAS	5.9	0.43
2B	Ler-Red-7WAS	14.5	1.17
2B	Ler-Red-8WAS	22.8	2.09
2B	Ler-Red-9WAS	32.3	2.96
2B	Ler-Red-10WAS	39.1	3.62
2B	Ler-Red-11WAS	41.2	3.90
2B	Ler-Red-12WAS	41.3	3.98
2E	MM-White-hypocotyl	3.2	0.11
2E	MM-White-epicotyl	2.8	0.28
2E	MM-White-stem	19.1	0.93
2E	MM-Red-hypocotyl	5.9	0.13
2E	MM-Red-epicotyl	7.3	0.49
2E	MM-Red-stem	25.7	0.97
2E	MM-Blue-hypocotyl	3.9	0.24
2E	MM-Blue-epicotyl	3.8	0.44
2E	MM-Blue-stem	24.5	0.95
2E	FO-White-hypocotyl	3.9	0.14
2E	FO-White-epicotyl	4.6	0.17
2E	FO-White-stem	33.3	1.11
2E	FO-Red-hypocotyl	7.1	0.22
2E	FO-Red-epicotyl	7.3	0.49
2E	FO-Red-stem	36.8	1.02
2E	FO-Blue-hypocotyl	4.2	0.26
2E	FO-Blue-epicotyl	5.1	0.58
2E	FO-Blue-stem	36.1	1.39
2F	MM-White-height-2WAS	2.5	0.07
2F	MM-White-height-4WAS	6.8	0.21

2F	MM-White-height-6WAS	25.1	0.92
2F	MM-Red-height-2WAS	4.2	0.08
2F	MM-Red-height-4WAS	11.5	0.31
2F	MM-Red-height-6WAS	36.1	0.75
2F	MM-Blue-height-2WAS	1.9	0.06
2F	MM-Blue-height-4WAS	7.2	0.23
2F	MM-Blue-height-6WAS	32.7	1.01
2F	FO-White-height-2WAS	3.0	0.10
2F	FO-White-height-4WAS	9.9	0.27
2F	FO-White-height-6WAS	41.8	1.12
2F	FO-Red-height-2WAS	5.5	0.23
2F	FO-Red-height-4WAS	16.5	0.62
2F	FO-Red-height-6WAS	51.2	1.17
2F	FO-Blue-height-2WAS	2.2	0.14
2F	FO-Blue-height-4WAS	7.8	0.41
2F	FO-Blue-height-6WAS	45.4	1.65
2F	MM-White-hypocotyl-2WAS	2.4	0.06
2F	MM-White-hypocotyl-4WAS	3.1	0.08
2F	MM-White-hypocotyl-6WAS	3.2	0.11
2F	MM-Red-hypocotyl-2WAS	4.2	0.07
2F	MM-Red-hypocotyl-4WAS	5.9	0.13
2F	MM-Red-hypocotyl-6WAS	5.9	0.13
2F	MM-Blue-hypocotyl-2WAS	1.9	0.05
2F	MM-Blue-hypocotyl-4WAS	3.6	0.11
2F	MM-Blue-hypocotyl-6WAS	3.9	0.24
2F	FO-White-hypocotyl-2WAS	2.9	0.09
2F	FO-White-hypocotyl-4WAS	3.8	0.08
2F	FO-White-hypocotyl-6WAS	3.9	0.14
2F	FO-Red-hypocotyl-2WAS	5.2	0.21
2F	FO-Red-hypocotyl-4WAS	7.0	0.19
2F	FO-Red-hypocotyl-6WAS	7.1	0.22
2F	FO-Blue-hypocotyl-2WAS	2.1	0.12
2F	FO-Blue-hypocotyl-4WAS	3.8	0.14
2F	FO-Blue-hypocotyl-6WAS	4.2	0.26
2F	MM-White-epicotyl-2WAS	0.1	0.02
2F	MM-White-epicotyl-4WAS	2.1	0.11
2F	MM-White-epicotyl-6WAS	2.8	0.28
2F	MM-Red-epicotyl-2WAS	0.1	0.02
2F	MM-Red-epicotyl-4WAS	3.4	0.13
2F	MM-Red-epicotyl-6WAS	4.4	0.36
2F	MM-Blue-epicotyl-2WAS	0.05	0.01
2F	MM-Blue-epicotyl-4WAS	2.3	0.13
2F	MM-Blue-epicotyl-6WAS	3.8	0.44
2F	FO-White-epicotyl-2WAS	0.2	0.02
2F	FO-White-epicotyl-4WAS	3.4	0.08
2F	FO-White-epicotyl-6WAS	4.6	0.17
2F	FO-Red-epicotyl-2WAS	0.2	0.03
2F	FO-Red-epicotyl-4WAS	5.6	0.21
2F	FO-Red-epicotyl-6WAS	7.3	0.49
2F	FO-Blue-epicotyl-2WAS	0.1	0.03
2F	FO-Blue-epicotyl-4WAS	2.7	0.16
2F	FO-Blue-epicotyl-6WAS	5.1	0.58
3B	Col-White-secondary	3.7	0.19
3B	Col-White-tertiary	11.5	0.76
3B	Col-Red-secondary	6.7	0.46

3B	Col-Red-tertiary	21.6	3.59
3B	Col-Blue-secondary	3	0.14
3B	Col-Blue-tertiary	4.4	0.24
3B	Ler-White-secondary	2.9	0.19
3B	Ler-White-tertiary	8.6	0.86
3B	Ler-Red-secondary	5.8	0.46
3B	Ler-Red-tertiary	17.5	3.12
3B	Ler-Blue-secondary	1.8	0.15
3B	Ler-Blue-tertiary	2	0.38
4D	Col-White-1WAS	2	0
4D	Col-White-2WAS	5.9	0.10
4D	Col-White-3WAS	10.3	0.18
4D	Col-White-4WAS	14.2	0.24
4D	Col-Red-1WAS	2	0
4D	Col-Red-2WAS	6.7	0.10
4D	Col-Red-3WAS	12.4	0.27
4D	Col-Red-4WAS	20.4	0.35
4D	Col-Red-5WAS	30.2	0.40
4D	Col-Red-6WAS	35.3	0.29
4D	Col-Red-7WAS	41.9	0.31
4D	Col-Red-8WAS	42.1	0.33
4D	Col-Blue-1WAS	2	0
4D	Col-Blue-2WAS	5.4	0.09
4D	Ler-White-1WAS	2	0
4D	Ler-White-2WAS	6.1	0.09
4D	Ler-White-3WAS	9.4	0.15
4D	Ler-Red-1WAS	2	0
4D	Ler-Red-2WAS	5.9	0.16
4D	Ler-Red-3WAS	11.8	0.22
4D	Ler-Red-4WAS	17.8	0.33
4D	Ler-Red-5WAS	27.1	0.47
4D	Ler-Red-6WAS	27.3	0.51
4D	Ler-Blue-1WAS	2	0
4D	Ler-Blue-2WAS	4.6	0.10
5C	MM-White-1WAS	0	0
5C	MM-White-2WAS	2	0
5C	MM-White-3WAS	3.3	0.12
5C	MM-White-4WAS	4.9	0.16
5C	MM-White-5WAS	7.5	0.11
5C	MM-Red-1WAS	0	0
5C	MM-Red-2WAS	2	0
5C	MM-Red-3WAS	3.3	0.13
5C	MM-Red-4WAS	4.8	0.09
5C	MM-Red-5WAS	7.4	0.14
5C	MM-Blue-1WAS	0	0
5C	MM-Blue-2WAS	2	0
5C	MM-Blue-3WAS	3.2	0.16
5C	MM-Blue-4WAS	4.9	0.13
5C	MM-Blue-5WAS	7.5	0.11
5C	FO-White-1WAS	0	0
5C	FO-White-2WAS	2	0
5C	FO-White-3WAS	3.2	0.12
5C	FO-White-4WAS	4.9	0.10
5C	FO-White-5WAS	7.5	0.14
5C	FO-Red-1WAS	0	0

5C	FO-Red-2WAS	2	0
5C	FO-Red-3WAS	3.1	0.11
5C	FO-Red-4WAS	4.8	0.11
5C	FO-Red-5WAS	7.4	0.15
5C	FO-Blue-1WAS	0	0
5C	FO-Blue-2WAS	2	0
5C	FO-Blue-3WAS	2.9	0.17
5C	FO-Blue-4WAS	4.4	0.26
5C	FO-Blue-5WAS	7.4	0.17
S1A	Ara-White-1DAS	87.5	3.78
S1A	Ara-White-2DAS	94.3	1.26
S1A	Ara-White-3DAS	94.3	1.26
S1A	Ara-Red-1DAS	70.3	5.91
S1A	Ara-Red-2DAS	91.2	2.71
S1A	Ara-Red-3DAS	95.6	1.99
S1A	Ara-Blue-1DAS	64.2	5.32
S1A	Ara-Blue-2DAS	90.1	2.99
S1A	Ara-Blue-2DAS	93.8	2.04
S1B	Tom-White-4DAS	28.6	4.37
S1B	Tom-White-5DAS	64.3	2.51
S1B	Tom-White-6DAS	92.9	1.77
S1B	Tom-Red-4DAS	31.2	3.94
S1B	Tom-Red-5DAS	50.0	2.92
S1B	Tom-Red-6DAS	87.5	1.83
S1B	Tom-Blue-4DAS	6.7	3.01
S1B	Tom-Blue-5DAS	33.3	2.67
S1B	Tom-Blue-6DAS	33.3	2.67
S1B	Tom-Dark-4DAS	100	0.92
S1B	Tom-Dark-5DAS	100	0.92
S1B	Tom-Dark-6DAS	100	0.92

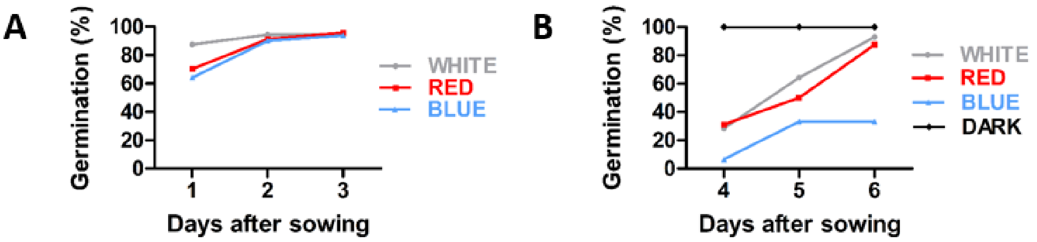

Figure S1: Arabidopsis and tomato germination assays.

A. Arabidopsis seeds showed optimal germination in white light. **B.** Tomato seeds showed optimal germination in darkness. Standard errors (from 3 technical replicates) are listed in **Table S2**.

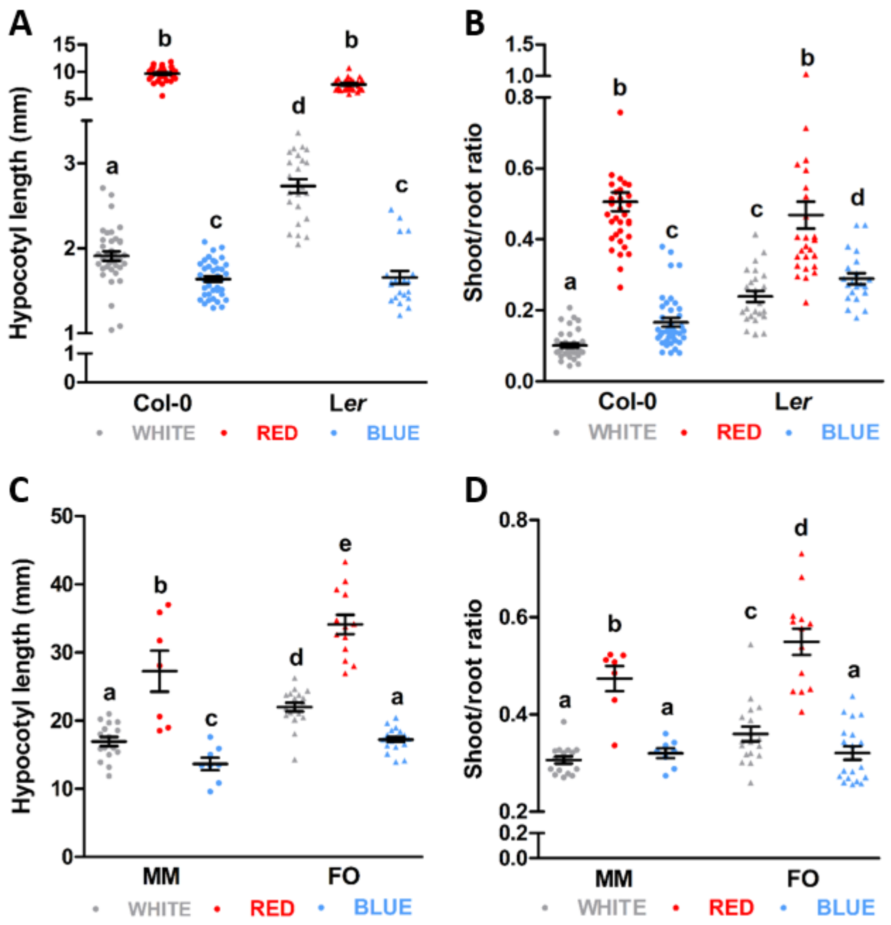

Figure S2: Quantification of Arabidopsis and tomato seedlings grown in light-grown roots (LGR) LED conditions.

A-B. Quantification of hypocotyl length (**A**) and shoot/root ratio (**B**) of 7 day old Arabidopsis seedlings of ecotypes Columbia (Col-0) and Landsberg *erecta* (L*er*) grown in white, red or blue LED light-grown roots (LGR) conditions. **C-D.** Quantification of the hypocotyl length (**C**) and shoot/root ratio (**D**) of 5 day old tomato seedlings of cultivars Moneymaker (MM) and Foundation (FO). LED conditions and ecotypes / cultivars were compared using a one-way ANOVA followed by a Tukey's test (letters **a**, **b**, **c**, **d**, and **e** indicate statistically significant differences, $p<0.05$). Error bars represent standard error of the mean in **A-B** (n=30) and **C-D** (n=20). Similar results were obtained in three independent experiments.

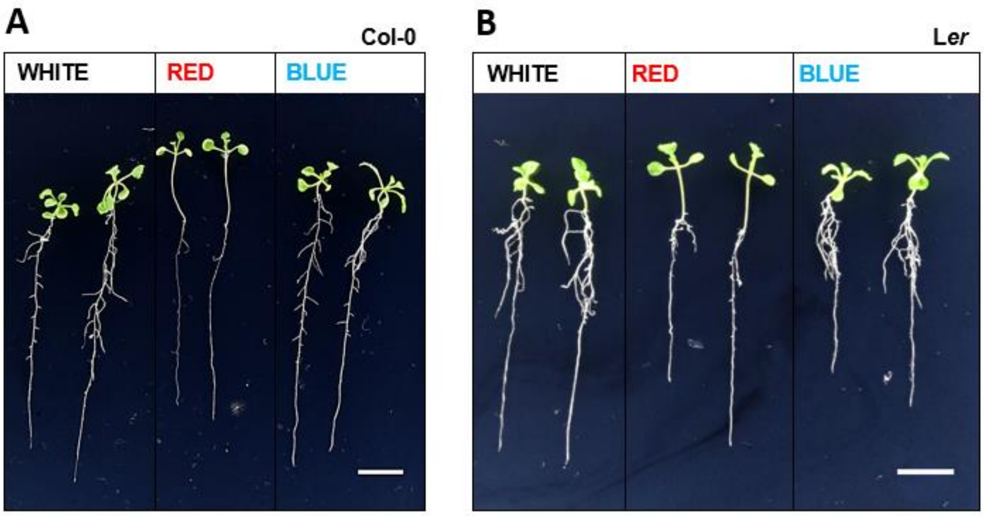

Figure S3: Phenotypes of 14-day old Arabidopsis seedlings.

A. Representative 14 day old Arabidopsis Columbia (Col-0) seedlings grown in dark-grown roots (DGR) white, red or blue LED conditions. **B.** Representative 14 day old Arabidopsis Landsberg *erecta* (L*er*) seedlings grown in dark-grown roots (DGR) white, red or blue LED conditions. For presentation purposes, seedlings were transferred to black agarose plates before photographing. Scale bars indicate 1 cm.

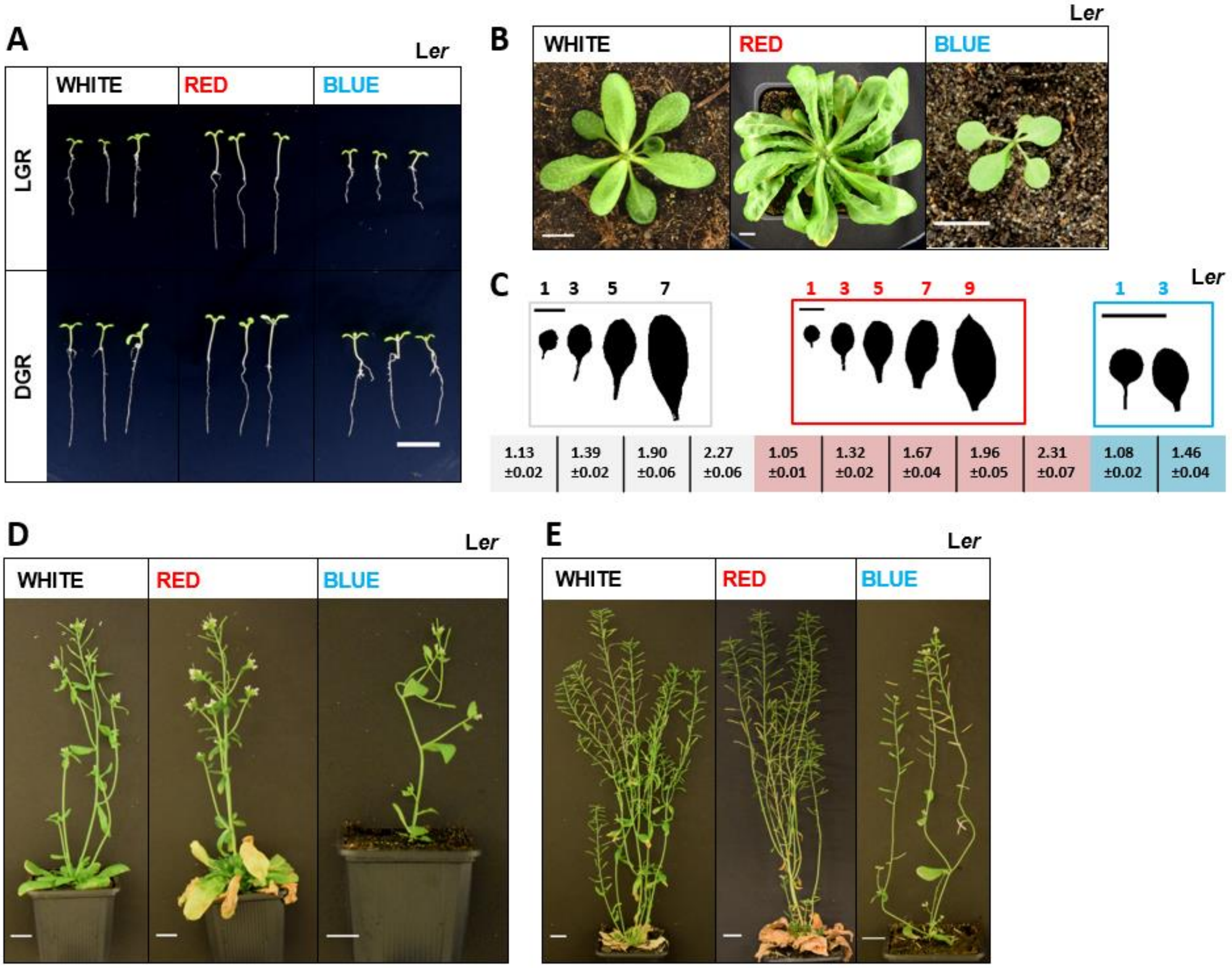

Figure S4: Phenotypes of Arabidopsis Landsberg *erecta* seedlings and plants grown under different LED lighting conditions.

A. Representative pictures of 7-day old Arabidopsis seedlings of ecotype Landsberg *erecta* (L*er*) grown in light-grown roots (LGR) or dark-grown roots (DGR) white, red, or blue LED conditions. For presentation purposes, seedlings were transferred to black agarose plates before photographing. **B.** Rosettes of representative L*er* plants grown in white, red, or blue LED conditions. **C.** Rosette leaves of representative L*er* plants and length/width ratios of the leaf blade (±SE). **D-E.** Representative L*er* plants at one week (**D**) or 4 weeks after flowering (**E**). Monochromatic LED conditions (red or blue) were compared to white (control) using a two-sided Student's *t*-test (asterisks indicate statistically significant differences, $p<0.05$) in **C** (n=10). Scale bars indicate 1 cm. Similar results were obtained in two (**A**) or three (**B-E**) independent experiments.

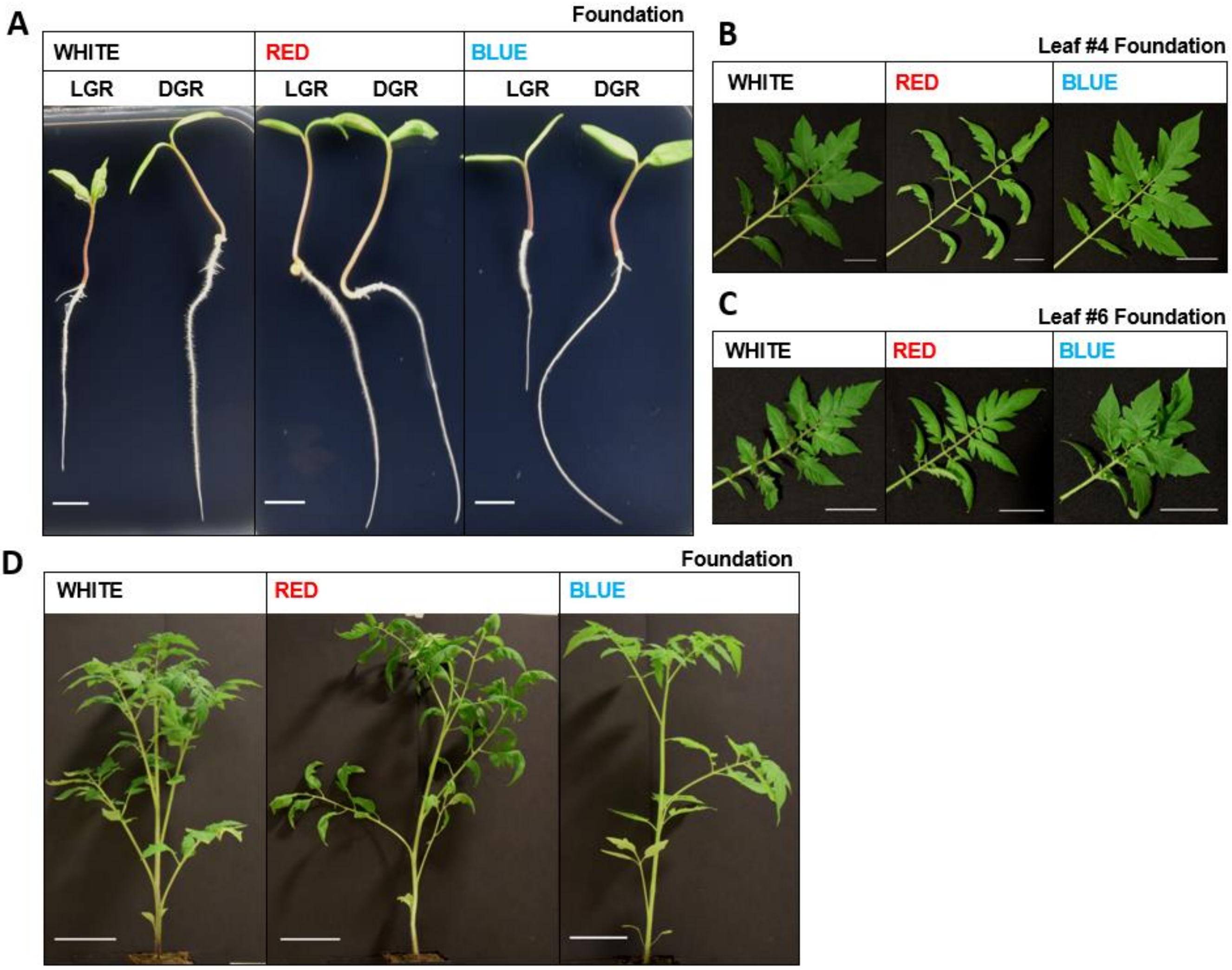

Figure S5: Phenotypes of tomato cultivar Foundation seedlings and plants grown under different LED lighting conditions.

A. Representative 5 day old tomato seedlings of cultivar Foundation (FO) grown light-grown roots (LGR) or dark-grown roots (DGR) conditions in white, red, or blue LED conditions. For presentation purposes, seedlings were transferred to black agarose plates. **B-C.** Representative compound leaves from FO plants grown in LED conditions (45 days after sowing (DAS)): leaf #4 (**B**), leaf #6 (**C**). **D.** Representative FO plants at 45 DAS. Scale bars indicate 1 cm in **A** and 5 cm in **B-D**. Similar results were obtained in two (**A**) or three (**B-D**) independent experiments.
